# SOCAV: a nurse-led support programme for self-direction in people with dementia receiving home care, involving informal caregivers – a feasibility study with process evaluation in the Netherlands

**DOI:** 10.1136/bmjopen-2025-105939

**Published:** 2026-03-18

**Authors:** Phébe Das, Gerbrich Douma, Hanneke Donkers, Lieve Roets-Merken, Maud Graff

**Affiliations:** 1 IQ Health, Radboud University Medical Center, Nijmegen, Netherlands; 2 Radboudumc Alzheimer Center, Nijmegen, Netherlands; 3 Rehabilitation, Radboudumc, Nijmegen, Netherlands

**Keywords:** Person-Centered Care, Dementia, Nursing Care, Feasibility Studies, Primary Care, Caregivers

## Abstract

**Abstract:**

**Objectives:**

To adapt the SOCAV programme—originally developed for residential dementia care—for home care use, and to evaluate its feasibility and potential to foster behavioural change in nurses and informal caregivers supporting self-direction in people with dementia.

**Design:**

Development and feasibility study guided by the Medical Research Council framework for complex interventions. Feasibility was evaluated using Bowen’s framework (demand, acceptability, practicality, implementation, limited efficacy). Data collection involved semistructured interviews, focus groups and reflective coaching diaries, as well as validated outcome measures (self-direction, quality of life and depressive symptoms) assessed at multiple time points. Qualitative data were analysed using qualitative content analysis (Bowen’s feasibility framework) and constant comparative analysis; quantitative data were analysed descriptively.

**Setting:**

Two home care teams in different Dutch municipalities.

**Participants:**

Development phase: 16 participants (4 people with dementia, 6 informal caregivers, 6 nurses). Feasibility phase: 59 participants (12 people with dementia, 14 informal caregivers, 33 nurses).

**Intervention:**

SOCAV-Home Care integrates person-centred communication training with reflective coaching for nurses and joint meetings involving people with dementia and informal caregivers. It aims to embed self-direction into daily care routines.

**Results:**

The programme was feasible and well-received, though demanding. Nurses reported increased reflection, more person-centred communication and greater professional confidence. Informal caregivers showed attitudinal shifts from control to autonomy-supportive care. Programme complexity, scheduling difficulties and emotional burden contributed to dropout. Quantitative trends showed reduced behavioural symptoms in people with dementia, though no statistical analysis was performed due to sample size.

**Conclusions:**

SOCAV-Home Care shows potential to foster behavioural change in nurses and informal caregivers, promoting self-direction and relational care in dementia home care. Findings, grounded in rich reflective data, offer a valuable foundation for further evaluation. Simplifying delivery and enhancing flexibility are key to broader implementation. Future research should evaluate the sustainability strategies proposed and examine long-term outcomes in diverse home care contexts.

**Trial registration number:**

NCT07347639; Post-results.

**STRENGTHS AND LIMITATIONS OF THIS STUDY**

- The SOCAV intervention integrates training, reflective coaching and collaborative home meetings to support self-direction in dementia home care.
- Feasibility was comprehensively assessed using Bowen’s framework, covering demand, acceptability, practicality, implementation and limited efficacy.
- Multiperspective data collection involving people with dementia, informal caregivers and nurses enhanced the contextual relevance of findings.
- Reflective diaries and focus groups indicated behavioural and attitudinal change among participants, suggesting potential for practice improvement.
- Programme complexity and participant burden contributed to partial dropout and limited sample size, restricting generalisability and long-term conclusions.

## Introduction

 Dementia significantly affects the daily functioning, autonomy and quality of life of people with dementia and their informal caregivers due to progressive cognitive and physical decline, behavioural changes and the resulting caregiver burden.^1^ People with dementia often face reduced participation in meaningful activities and social interactions, along with the psychological burden of losing independence and facing an uncertain future.^2 3^

For informal caregivers, particularly partners, this results in emotional and practical challenges as relational dynamics shift from equal partnerships to care-dependent relationships. These changes disrupt relational patterns, leading to emotional strain, grief and an overwhelming sense of responsibility.^4 5^ The progressive loss of self-direction in people with dementia increases reliance on informal caregivers, which further reduces quality of life for both.^6 7^

These challenges highlight the urgent need for interventions that support the preferences, self-direction and dignity of people with dementia, while also alleviating caregiver burden. Over recent decades, dementia care has shifted from task-oriented approaches to person-centred and relationship-centred models, emphasising the unique needs, preferences and relationships of people with dementia and those who care for them.^8 9^ Person-centred care treats individuals as people with unique life stories and preferences, while relationship-centred care builds on this foundation by fostering meaningful connections between people with dementia, informal caregivers and professionals to support well-being.^10^

Together, these approaches offer a platform for preserving self-direction, which in the Dutch care and treatment context has gained prominence as an essential extension of autonomy. It is increasingly seen not as an individual ability but as a dynamic, relational process shaped by daily interactions.^11^ Self-direction refers to the capacity of people with dementia to participate in everyday decisions and express preferences, not necessarily independently, but with the support of informal caregivers and professionals who clarify and act on those preferences. This relational view recognises that self-direction remains possible amid cognitive decline, provided the social environment enables and respects the person’s agency. Implementing this view requires real-world interaction, where caregivers balance support with allowing control, even in small daily matters like meals or routines. Yet as dementia progresses, safety and convenience often take precedence, limiting opportunities for self-direction.^12^

Across international dementia care systems, there is growing recognition that autonomy and self-direction are essential to preserving dignity and well-being. These values are embedded in person-centred and relationship-centred frameworks, which are increasingly adopted as guiding principles in dementia care policy and practice.^13^ However, translating these principles into real-world home care remains challenging. Research highlights that enabling self-direction requires not only ethical intent but also specific knowledge, communication skills and relational competence to support everyday decision-making in dynamic caregiving contexts.^14^ Moreover, the lack of structured, evidence-based approaches in this setting limits caregivers’ ability to act on preferences consistently.^15^ Interventions like SOCAV—a nurse-led support programme originally developed to enhance self-direction in residential dementia care—may therefore contribute to internationally relevant insights into how self-direction can be supported effectively in diverse home care systems.

Several international studies highlight that enabling self-direction in practice demands specific knowledge, skills and attitudes. These include knowing the person’s life story and preferences and enabling their role in decision-making and goal setting.^16^,^19^ Training helps caregivers balance risk and autonomy. Unlike reablement, which targets functional restoration,^20^ supporting self-direction focuses on maintaining meaningful participation and personal well-being. Despite this understanding, evidence-based person-centred and relationship-centred interventions remain scarce—especially in home care, where relational and contextual variability pose distinct challenges.^7 21^ Tailored, scalable solutions are urgently needed to support self-direction in this setting.

The SOCAV programme, originally developed for residential care, addresses this gap by integrating two complementary approaches: the evidence-based Community Occupational Therapy in Dementia (COTiD), designed to improve daily functioning,^16 17 22^ and the Kalorama Reflective Coaching (KRC) approach, which fosters critical reflection among care professionals.^23 24^ SOCAV combines person-centred communication training with longitudinal coaching to enhance self-direction in people with dementia and to strengthen the communication skills of both professionals and informal caregivers. The programme has demonstrated its potential in nursing home settings to improve reflective practice, helping caregivers balance safety with self-direction and align their behaviours with person-centred care principles.^25^ Encouraged by these findings, this study adapted SOCAV for home care to address the specific challenges of supporting self-direction in community-dwelling people with dementia.

The research questions were as follows: (1) What adaptations are needed to make the SOCAV residential care programme suitable for application in home care? (2a) What is the feasibility of this modified SOCAV-Home Care programme in terms of demand, acceptability, practicality, implementation and limited efficacy? (2b) What is the potential of this modified SOCAV-Home Care programme to align the attitudes and behaviours of informal caregivers and professionals with person-centred care principles?

## Methods

### Study design

This study followed the first two phases of the Medical Research Council framework for complex interventions: development and feasibility.^26 27^ In the development phase, qualitative data from interviews and prototype testing were used to inform programme adaptations. The feasibility phase evaluated Bowen’s five domains: acceptability, demand, practicality, implementation and limited efficacy,^28^ using primarily qualitative methods, supplemented by limited quantitative measures to assess feasibility.

### Setting and participants

Three stakeholder groups participated: people with dementia, their informal caregivers and home care nurses, all affiliated with a regional Dutch home care organisation. The development phase took place in one mid-sized municipality, the feasibility phase in a neighbouring area. People with dementia were eligible if they had mild to moderate dementia diagnosed by a general practitioner or geriatrician,^29^ lived in the community and received care from a home care team. Those with a Geriatric Depression Scale score >6 were excluded.^30^ Informal caregivers were eligible if they provided care at least twice a week, either as live-in (primary) or regularly visiting (secondary) caregivers. Nurses were eligible if they worked in home care, supported people with dementia and were employed by the regional care organisation.

Recruitment was facilitated by a care manager who provided verbal information and obtained written informed consent from people with dementia and their informal caregivers. Nurses joined as part of their employer’s regular professional development training; written consent was not required, but participation was voluntary, and procedures were explained. Training and coaching were delivered during working hours.

Interviewers (PD and GD) were trained researchers with experience in dementia care but independent of participants’ care. They were unknown to participants and were introduced by a familiar care professional, who clarified that they were independent and affiliated with a university medical centre.

### SOCAV programme

The original SOCAV residential care programme is a training and coaching intervention designed to enhance person-centred care and self-direction support for people with dementia in nursing home care settings. This study focused on its adaptation to the home care setting, referred to as SOCAV-Home Care (SOCAV-HC).

The programme integrates two evidence-based approaches: the COTiD programme and the KRC method. The COTiD programme promotes self-direction, daily functioning and quality of life for people with dementia through person-centred communication and observation, shared goal setting, meaningful activity engagement and environmental adaptations tailored to individuals’ abilities. It also enhances informal caregiver competence by offering practical strategies and enhancing their problem-solving capacity.^16 22 31^ The KRC method focuses on reflective coaching to help nurses develop person-centred attitudes and behaviours. It emphasises observing the needs and preferences of people with dementia and encourages nurses to align their practices with the preferences of people with dementia, and their self-direction and problem-solving abilities.^24^

Together, these programmes form the foundation of the SOCAV programme’s structured training and coaching approach. Table 1 presents the core components of the SOCAV-Home Care programme, distinguishing between the original components and the adaptations made to tailor the intervention to the home care context.

**Table 1:** Core components of the SOCAV-Home Care programme, including the original modules and supplemental components added following the study findings

Phase	Description	Details
Phase 1—Training of SOCAV Peer Coaches (Original module)	Audience: Peer coaches Delivered by: COTiD and KRC experts	Person-centred communication Observation of strategies, abilities and well-being Shared goal setting and reflective interviewing *Format: 5 sessions (2 hours), over 4 months*
Phase 2a—Training of nursing staff (Original module)	Audience: Nurses Delivered by: SOCAV-trained peer coach	Dementia education and self-directed care Person-centred communication (COTiD) Simulation session using an ageing suit and virtual reality goggles Workshop: identify behaviours that support/reduce self-direction *Format: 4 sessions (2 hours) +1 workshop (1 hour), over 6 weeks*
Phase 2b—Introduction for informal caregivers (Supplemental module)	Audience: Informal caregivers Delivered by: SOCAV-trained peer coach	Understanding dementia-related changes Promoting person-centred attitudes and self-direction Shared goal setting and functional observation *Format: 3 sessions (1.5 hours), over 6 weeks*
Phase 3a—Reflective coaching of nursing staff (Original module)	Audience: Nurses Delivered by: SOCAV-trained peer coach	One-on-one reflection on care experiences Use of reflective diaries to define learning goals Team-based peer reflection sessions *Format: 2 intro+8 individual sessions (30 min), 4 group sessions (2 hours), over 6–9 months*
Phase 3b—Home meetings (Supplemental module)	Participants: Person with dementia, informal caregiver, nurse, and peer coach	Mapping preferences and daily routines Shared goal setting and strategy evaluation Empowering caregivers through reflective dialogue *Format: 4–6 meetings (1–1.5 hours), over 3–6 months*

Phases labelled as ‘original module’ refer to components of the initially implemented programme; phases labelled as ‘supplemental module’ were added following the findings in the present study.

COTiD, Community Occupational Therapy in Dementia.

### Implementation and delivery

Two peer coaches—an experienced nurse and a social worker from the regional home care organisation—were selected through an open call based on their dementia care experience, communication skills, and readiness to engage in structured reflective practice. They received specialised training from a COTiD therapist and a KRC psychologist and subsequently delivered the SOCAV programme to their colleagues. Their responsibilities included facilitating individual and group coaching sessions, modelling reflective practice and supporting nurses in applying person-centred care principles in daily practice. Peer coach training consisted of five sessions (2 hours each) over 4 months, combining COTiD elements (person-centred communication, observation of daily functioning, shared goal setting) with KRC elements (observation of well-being, awareness of caregiver impact, and use of reflective questions). During implementation, the peer coaches received ongoing support from PD and LR-M through monthly check-ins and on-demand consultation.

For nurses, the full implementation period ranged from 6 to 9 months, during which training, coaching and application in daily practice took place in parallel. They participated in a series of training sessions and engaged in longitudinal peer coaching to strengthen their skills and confidence in delivering person-centred care and improving their practices in supporting self-direction. To support this process, nurses kept reflective diaries—either paper-based or digital—which served as the basis for individual coaching. Peer coaches facilitated both individual and group coaching sessions and guided nurses in the use of these diaries. Reflective diaries were introduced during the initial coaching sessions, and nurses were instructed to record short reflections on real care situations in which they supported a person’s self-direction—including what they did, what worked well and what they might do differently. To support consistent and meaningful reflection, peer coaches provided a short set of guiding prompts and examples of concise entries.

### Patient and public involvement

Patient and public involvement was integrated throughout the study’s design, development and feasibility phases. In the design phase, client representatives and non-participating home care nurses reviewed the draft protocol and advised on relevance, feasibility, ethics and outcome selection, informing recruitment and data collection procedures. During development, people with dementia and informal caregivers participated in interviews and focus groups to co-shape the SOCAV–Home Care prototype. In the feasibility phase, nurses, informal caregivers and peer coaches provided ongoing feedback through focus groups, reflective diaries and written reports, guiding refinements in programme delivery and coaching strategies. Although participants were not involved in formal data analysis, their input informed the adaptation of interview guides and the thematic framework, ensuring findings reflected lived experience. This section follows the GRIPP2 Short Form checklist^32^

### Data collection

During the development phase, qualitative data were collected at baseline through semistructured, in-person interviews with people with dementia, focus groups with informal caregivers, and separate focus groups with nurses. Prototype experiences were continuously monitored through reflective diaries kept by nurses, and at two time points (2 and 5 months) through focus groups with informal caregivers, and separate focus groups with nurses. Feedback was regularly gathered from the peer coaches but not at predetermined intervals. In addition, quantitative data were collected at baseline and after 5 months (month 5) to assess the suitability of the selected outcome measures and data collection procedures.

The feasibility phase, conducted in a different municipality and with a new group of participants, involved the collection of qualitative data throughout the approximately 9-month intervention period. Experiences and developments were continuously monitored through reflective diaries kept by nurses. At three time points—baseline, midpoint (around month 4), and endpoint (around month 9)—focus groups with informal caregivers and separate focus groups with each of the two nursing teams were conducted. Feedback was regularly gathered from the peer coaches, but not at predetermined intervals. Primary quantitative data were collected at all three time points, while secondary data were collected at baseline and at endpoint (month 9).

Interview guides were semistructured and iteratively refined after each set of interviews or focus groups, based on emerging themes, unclear or unproductive questions and interviewer observations. Revisions were discussed within the core research team (PD, GD, HD, LR-M and MG) to ensure relevance, clarity and alignment with the study objectives (see online supplemental file 1: Interview guide SOCAV-HC).

All interviews and focus groups were audio-recorded with participants’ consent and transcribed verbatim, anonymised and checked for accuracy by the research team. Each interview and focus group session lasted approximately 60 min. For all interviews with people living with dementia, an informal caregiver was present to provide emotional and practical support and to facilitate communication where needed.

**Figure 1:**
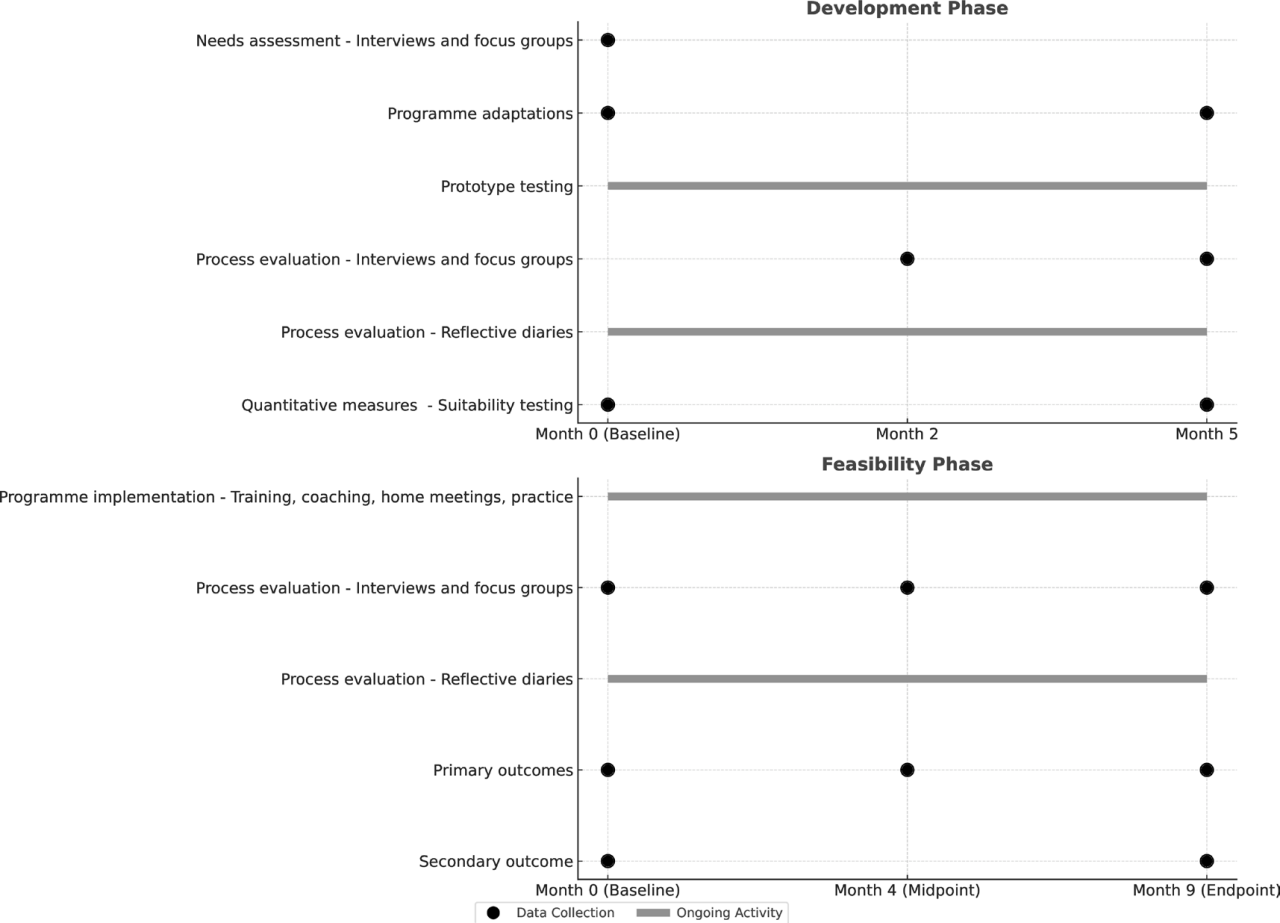
Study activities and data collection timeline.

Figure 1 shows the study timeline, including the sequence of study activities and scheduled data collection time points across the development and feasibility phases.

### Outcome measures

During the development phase, we evaluated the suitability of quantitative outcome measures and data collection procedures for this population and setting. This resulted in the selection of three primary outcome domains to explore limited efficacy during the feasibility phase: self-direction in people with dementia, quality of life and depressive symptoms in informal caregivers. Self-direction was assessed using the Dutch version of the Canadian Occupational Performance Measure (COPM), which evaluates perceived performance and satisfaction in meaningful activities on a 1–10 scale (1=not at all able/satisfied; 10=completely able/satisfied) based on reports from people with dementia, informal caregivers and nurses. The COPM has demonstrated adequate content and construct validity, feasibility in older adults and moderate responsiveness. Quality of life was assessed using the 29-item Dementia Quality of Life (DQoL-29) instrument, suitable for people with mild to moderate dementia. Depressive symptoms in informal caregivers were measured using the Centre for Epidemiologic Studies Depression Scale (CES-D), which shows strong internal consistency (Cronbach’s alpha=0.84) and reproducibility (r=0.71). Secondary outcomes included mood (Cornell Scale for Depression in Dementia, CSDD), behavioural symptoms (Neuropsychiatric Inventory Questionnaire, NPI-Q) and (The Older Persons and Informal Caregivers Survey - Minimum Data Set (TOPICS-MDS)).^3133^,^38^ Quantitative data were collected at predefined time points across the development and feasibility phases (table 2).

**Table 2:** Outcome measures, respondents and timing of assessment in the development and feasibility phases

Outcome domain	Outcome measure	Respondent	Development phase		Feasibility phase		
			Baseline–suitability testing	5 months–suitability testing	Baseline	4 months–midpoint	9 months–endpoint
Primary Self-direction	Canadian Occupational Performance Measure	Person with dementia; informal caregiver; nurse	✓	✓	✓	✓	✓
Primary Quality of life	Dementia Quality of Life-29	Person with dementia	✓	✓	✓	✓	✓
Primary Depressive symptoms caregiver	Centre for Epidemiologic Studies Depression Scale	Informal caregiver	✓	✓	✓	✓	✓
Secondary Mood	Cornell Scale for Depression in Dementia	Person with dementia	✓	✓	✓		✓
Secondary Behavioural symptoms	Neuropsychiatric Inventory Questionnaire	Informal caregiver	✓	✓	✓		✓
Secondary Quality of life caregiver	TOPICS-MDS	Informal caregiver	✓	✓	✓		✓

TOPICS-MDS, The Older Persons and Informal Caregivers Survey - Minimum Data Set.

### Data analysis

Qualitative data from interviews and focus groups were analysed using qualitative content analysis, guided by Bowen’s feasibility framework,^28^ with inductive coding within and across domains and informed by Corbin and Strauss’s analytic procedures.^39^ Analyses were conducted in Atlas.ti V.22.0^40^ through iterative reading, open coding and clustering of meaning units. Reflective coaching diaries and end-of-programme focus group data were analysed separately using a constant comparative approach with open and axial coding, informed by Corbin and Strauss, to identify shared and divergent insights across data sources. Codes were developed inductively and refined through repeated team discussions; no formal codebook was used. Data saturation was monitored, with no new categories emerging after the final transcripts were coded. Transcripts or interpretations were not returned to participants, to minimise burden and ensure feasibility.

Quantitative data were analysed descriptively (IBM SPSS V.27.0). Analysis of covariance was initially planned to assess changes in primary and secondary outcomes, but this was not feasible due to sample size and data completeness constraints.^41^

The core research team included health professionals and qualitative researchers with backgrounds in nursing, occupational therapy, psychology and dementia care. While these backgrounds provided relevant expertise and familiarity with the study context, we acknowledge that they may have influenced data interpretation. PD and LR-M led coding, with regular team discussions to validate interpretations and agree on coding frameworks and emerging categories. The interviewers were not involved in the participants’ care. Analytic decisions were discussed in team meetings and documented to ensure transparency.

Reporting adhered to the Consolidated Criteria for Reporting Qualitative Research checklist.^42^

## Findings

### Baseline characteristics

Table 3 presents the baseline characteristics of participants in both the development and feasibility phases.

**Table 3:** Baseline characteristics of people with dementia, informal caregivers and nurses in the development and feasibility phases

Characteristics	Development phase	Feasibility phase
**Persons with dementia**	**N=4**	**N=12**
Age, mean (range), years	83 (72–88)	84 (74–95)
Female, n (%)	2 (50%)	9 (75%)
Dementia severity, n (%)		
Mild	0 (0%)	5 (42%)
Moderate	3 (75%)	6 (50%)
Severe	1 (25%)	1 (8%)
Mood–CORNELL, mean (range)	11.0 (4–18)	7.6 (0–14)
**Informal caregivers**	**N=6**	**N=14**
Age, mean (range), years	74 (57–84)	62 (47–84)
Female, n (%)	5 (83%)	11 (79%)
Caregiving role, n (%)		
Primary caregiver	1 (25%)	3 (21%)
Secondary caregiver	3 (75%)	11 (79%)
Mood (CES-D), mean (range)	16.3 (8–29)	5.9 (0–28)
**Nurses**	**N=6**	**N=33**
Registered nurses, n (%)	1 (17%)	7 (21%)
Licensed nursing assistants, n (%)	5 (83%)	26 (79%)

CES-D, Centre for Epidemiologic Studies Depression Scale; CORNELL, Cornell Scale for Depression in Dementia.

### Development phase: needs, perspectives on self-direction and intervention adaptation

Four individuals with dementia receiving home care were interviewed. Focus groups were held with four informal caregivers (one primary, three secondary) and six nurses. People with dementia emphasised that stable and continuous routines were crucial for maintaining self-direction. They viewed stability as indirectly supporting self-direction by reducing disruptions and creating predictability. One participant summarised this sentiment by saying, *‘Life as it is now, is fine.’* Stability was seen as indirectly supporting self-direction by minimising disruption and ensuring predictability.

Informal caregivers described their role as an ongoing emotional and practical burden, intensified by the unpredictable nature of dementia. They often felt overwhelmed by the constant stream of decisions and challenges. When asked whether and how they supported their loved ones’ self-direction, they often responded with hesitation or scepticism. One informal caregiver remarked, “*I let my father cycle outside; let’s just hope he doesn’t fall”* (RMz03). Another, more cynically, said, “*When he’s at the day care centre and wants to go home, he’s not allowed. So, I guess that means self-direction is over, right?*” (RMz01).

Informal caregivers commonly viewed nurses’ roles as limited to practical tasks. One participant commented*, “They come to do the washing” (RMz04*). This minimal interaction reinforced informal caregivers’ sense of isolation and sole responsibility.

Nurses acknowledged that their interactions with people with dementia offered valuable insights into behavioural changes but reported minimal coordination with informal caregivers. They described their roles as predominantly task-focused and did not actively support self-direction. Many lacked awareness of the preferences of the people with dementia or the challenges faced by informal caregivers. As one nurse put it, supporting self-direction was ‘not on the radar’ and no structured methods or tools were available.

These findings revealed a critical gap in collaboration and shared understanding. While informal caregivers experienced a lack of support in balancing self-direction and safety, nurses lacked the training and resources to contribute to self-direction effectively.

To address these needs, wishes and contextual gaps, a prototype version of the SOCAV programme for home care was developed and refined through three adaptations. First, two components from the COTiD programme were added: the Occupational Performance History Interview (OPHI-II) and an ethnographic interview. The OPHI-II explores the lived experiences, goals and challenges of people with dementia; the ethnographic interview focuses on informal caregiver needs and experiences. Both were conducted by an independent occupational therapist not involved in the study.

Second, informal caregivers were invited to join the nurse training sessions, gaining dementia-specific knowledge and practical strategies to support self-direction.

Third, regular collaborative home meetings were introduced, bringing together people with dementia, informal caregivers and nurses. Facilitated by nurses with support from a SOCAV peer coach, these meetings focused on identifying needs and enhancing self-direction within the home context.

### Development phase: prototype testing

A prototype of the SOCAV programme, incorporating these adaptations, was piloted over 5 months in a mid-sized municipality in the south of the Netherlands, involving four dyads of people with dementia and informal caregivers, along with six nurses. Two dyads withdrew due to the death of one participant and the relocation of another. Despite careful planning, several components of the data collection process proved difficult to execute in full, primarily due to external constraints. Nonetheless, four OPHI-II interviews, four ethnographic interviews, two collaborative home meetings and eleven reflective coaching diaries were successfully collected.

Analysis of all these collected data, guided by Bowen’s feasibility criteria, showed that the programme’s complexity posed significant challenges for informal caregivers, particularly in the coordination of training sessions, coaching, home visits and outcome measure assessments. Nurses, meanwhile, struggled to grasp the purpose of reflective coaching, often expecting the peer coach to provide feedback or correction rather than support reflective thinking.

Based on these lessons, three key adjustments were made to the SOCAV programme. The OPHI-II and ethnographic interviews were integrated into the collaborative home meetings to streamline the process. The joint training for informal caregivers and nurses was replaced with a shorter, tailored training specifically for informal caregivers. Additionally, two introductory coaching sessions were added for nurses to familiarise them with the reflective coaching approach in a supportive and non-judgemental manner.

### Feasibility phase

Following the prototype adjustments, the adapted SOCAV-Home Care programme was implemented in a neighbouring municipality as part of the feasibility phase. Twelve people with dementia participated in this phase, alongside 14 informal caregivers and 33 nurses. Data were collected via 12 individual interviews with people with dementia, 16 interviews with informal caregivers, 2 focus groups with informal caregivers, 2 with nurses and 281 reflective diaries completed by nurses. All participating nurses, along with 5 of the 12 dyads of people with dementia and their informal caregivers, completed the full programme. In seven dyads, one member withdrew during the study: in four cases the person with dementia either passed away or became emotionally distressed; in the remaining three, informal caregivers cited medical complications or felt that the programme was either too demanding or not personally relevant.

Feasibility was first examined through content analysis of qualitative data from interviews with people with dementia and focus groups with informal caregivers and nurses, guided by Bowen’s framework, which encompasses demand, acceptability, practicality, implementation and limited efficacy. Feasibility was further explored using constant comparison of data from end-of-programme focus groups and reflective coaching diaries completed by nurses.

#### Bowen’s feasibility aspects

##### Demand

Nurses expressed initial motivation to support self-direction but reported difficulties in applying this in practice. Early reflections often referred to external factors, such as limited time and perceived behaviour of people with dementia and their informal caregivers, rather than their own practices. Some also felt challenged by conflicting expectations between themselves and informal caregivers. Through coaching and structured home meetings, nurses reported that they learnt to better support the person with dementia’s self-direction and to respond more flexibly to varying demands.

Informal caregivers also identified needs, but before training, these were described in general terms. While they expressed the importance of self-direction, many were unclear about how to operationalise it. After training, informal caregivers developed a more concrete understanding of SOCAV goals and were able to articulate their own support needs more clearly. This shift was often accompanied by a stronger commitment to supporting autonomy.

##### Acceptability

Initially, informal caregivers expressed insecurity about the feasibility of supporting the wishes of people with dementia and about having to reflect on their own behaviour.

When they ask my mother what she wants, she comes up with unrealistic goals and answers like she wants to go on a vacation… we can’t fulfil that. (ElMz08)

As the programme progressed, discussing concrete situations with nurses helped informal caregivers feel more at ease with the idea of enabling self-direction. Nurses also initially experienced discomfort with reflective practice but gained confidence and insight through repeated coaching. Occasionally, tensions arose when other professionals involved in the care approached situations differently, as illustrated by a peer coach:

During the home meetings, we slowly worked on setting up a visit to a daycare centre and looked for suitable options, and a day later the case manager evoked all resistance around this topic by asking just one question. (SOCAV peer-coach).

##### Practicality

The number and intensity of SOCAV-related activities contributed to perceived burden among informal caregivers, occasionally resulting in dropout. Nurses considered digital coaching more time-efficient during periods with high workload, though sometimes less personal. Coordinating schedules with multiple informal caregivers proved challenging, but feasibility improved when individual appointments and digital sessions were used.

##### Implementation

Informal caregivers and nurses valued the support from SOCAV coaches in building confidence and learning to reflect on behavioural change. Caregivers found the training particularly useful. Both nursing teams expressed a desire to continue coaching in the future, though at a reduced frequency. One coach observed that the success of the programme depended less on educational background and more on intrinsic motivation and openness to behaviour change:

I would prefer to be able to have a chat with the SOCAV peer-coach in the future. She helps me get back on track. (ElVz11).

##### Limited efficacy and exploratory outcomes

Due to the small sample size and disruptions related to staffing constraints and care delivery pressures, no statistical analyses were conducted. Descriptive quantitative data revealed no noticeable changes in self-direction (COPM), quality of life (DQoL), informal caregiver depressive symptoms (CES-D), or mood (CSDD). However, exploratory trends were observed in behavioural symptoms, based on the NPI-Q. At baseline (N=11), all 12 neuropsychiatric symptoms included in the NPI-Q were present in the sample, with each symptom reported in at least one participating person with dementia. By the final assessment (N=5), only seven of these symptoms were still observed in the group. Notable reductions were seen in agitation, apathy, disinhibited behaviour and aimless repetitive behaviour. Although these findings were not statistically assessed, due to limited sample size, they may reflect clinically meaningful changes and support further investigation in a larger, adequately powered trial.

### Evaluation of attitude and behavioural change in informal caregivers and nurses

Constant comparison of end-of-programme interview data from informal caregivers and focus groups with nurses, supplemented by nurses’ reflective diaries, revealed meaningful shifts in how both groups supported self-direction. Among informal caregivers, two core categories of attitudinal change emerged: (1) a shift from a predominantly safety-focused mindset to a more relaxed, autonomy-supportive approach and (2) a transition from control towards enabling self-direction. Informal caregivers reported that participation in the training and home meetings enhanced their understanding of dementia and increased their confidence in setting boundaries and articulating personal limits. These changes were based on caregivers’ increased ability to reflect on their own behaviours and recognise ways to foster autonomy in everyday care. Table 4 presents the qualitative categories along with illustrative quotations.

**Table 4:** Qualitative categories illustrating changes in attitudes and behaviours among informal caregivers and nurses

Category	Description	Illustrative quotations
Caregivers—Towards a more relaxed attitude	Increased confidence led caregivers to shift from prioritising safety to fostering self-direction, while becoming more aware of their own behaviour.	*“I now know better where we are with our mother in the process of dementia.” (ElMz01)* *“I now realise I often asked those nagging questions, like I was testing or pestering her.” (ElMz04)* *“I thought, oh no, I’m doing this wrong. But I realised that myself.”* *“I do feel like I’ve become more tolerant.”* *“We’ve definitely gotten better at it.”*
Caregivers—Towards less control and more self-direction support	Caregivers initially focused on practical support. Post-training, they articulated needs more clearly, gained dementia knowledge, and promoted self-direction.	*“When arriving, I always immediately looked in the fridge or started making coffee.” (ElMz01)* *“We actually never really talked about autonomy with him.”* *“I don’t take as much out of his hands anymore. I noticed that he can do more on his own.”* *“I did learn something about how to ask questions.”* *“It’s purely about how I reflected on my own behaviour.”*
Nurses—Managing professional control	Nurses began shifting from task-oriented to personalised care by observing and engaging individuals with dementia.	*“At the start, I sometimes caught myself just taking clothes out… later asking: what do you want to wear?” (ElVz04)* *“You want to do things well, influencing and pre-chewing everything for the client.”* *“Without SOCAV, I would offer the solution. With SOCAV, I let the client think along.”*
Nurses—Exploring self-direction and co-direction	Nurses evolved from controlling behaviour to enabling individuals with dementia to participate in decisions.	*“Don’t act right away… More: How could you or we solve that?” (ElVz19)* *“Let them come up with something themselves. That was an eye-opener.”* *“Doing this together with others, purely sparring a bit.”*
Nurses—Discovering the power of reflection and interaction	Reflective coaching enabled nurses to identify how routines limited autonomy and adjust care accordingly.	*“I believe it would help to engage collectively, exchange ideas, and reflect together.”*
Nurses—Gaining confidence in one’s own professionalism	Nurses developed confidence in reflection and appreciated insights from informal caregivers, recognising the need for continued dialogue.	*“I find it valuable to hear informal caregivers’ perspectives.”* *“I’m curious about how informal caregivers view things, as their perspective isn’t always shared.”*

Nurses described a similar developmental trajectory in supporting self-direction. Reflective coaching enabled them to recognise and adjust routine behaviours that inadvertently restricted opportunities for self-direction among people with dementia. Over time, many nurses reported a shift from directive care to a more collaborative, shared decision-making approach. Constant comparison of the reflective diaries revealed four interrelated categories that underpinned this attitudinal and behavioural change: (1) managing professional control, (2) exploring self-direction and co-direction, (3) discovering the power of reflection and interaction and (4) gaining confidence in one’s own professionalism. Although most nurses expressed increased confidence in applying person-centred principles, several emphasised the need for ongoing coaching to maintain and deepen these attitudinal and behavioural changes over time.

### Additional insights from reflective coaching diaries

In addition to attitudinal change, nurses’ reflective diaries revealed a shift in the content of day-to-day conversations with people living with dementia. These interactions moved beyond routine physical care to include new and often unexpected topics, many of which were initiated by the clients themselves. This change reflected a broader understanding of the nursing role—one that included space for personal meaning, emotional expression and shared reflection.

During the development phase, both informal caregivers and nurses primarily described nursing support as practical and task-oriented, focusing on bodily care such as washing and dressing. While the SOCAV-Home Care programme encouraged more person-centred conversations, no structured method for documenting conversation content was included in the study design. Therefore, the topics that emerged in the reflective diaries arose organically from everyday interactions.

In an exploratory, post hoc categorisation of the 281 diary entries, we identified 150 distinct conversation topics. Many of these were introduced by the people with dementia and covered a wide range of issues—from organisational matters such as scheduling or household responsibilities to deeply personal and emotional themes, including loneliness, anxiety, and changing family roles (see online supplemental material 2: Conversation Topics Table from the SOCAV-Home Care Study). These findings suggest that nurses became more open to following the conversational lead of their clients, allowing care interactions to evolve into more relational and reflective exchanges.

## Discussion

This study supports the feasibility of the SOCAV-Home Care (SOCAV-HC) programme in fostering self-direction among people with dementia living at home. The intervention was perceived to facilitate meaningful attitudinal and behavioural change in nurses and informal caregivers, enhancing their capacity to promote person-centred and relationship-based care. Guided by Bowen’s framework, the evaluation demonstrated strong demand, acceptability, and practicality, while also identifying challenges in programme complexity and implementation in home-based contexts. These observed changes appeared to result from an evolving understanding of self-direction, developed through experiential learning, reflective coaching and collaborative home meetings. Over time, participants reported growing confidence in engaging people with dementia in everyday choices and rethinking their own routines to better support individual preferences.

Given the small sample size and the exclusive reliance on qualitative data—reflective diaries, interviews and focus groups—the findings should be interpreted as indicators of feasibility. Although participants described meaningful changes, the extent to which these were enacted in practice warrants further investigation.

### Strengths and limitations

A key strength of this study is its integration of person-centred training, collaborative home meetings and reflective coaching within a longitudinal, real-world setting. Reflective coaching, delivered by a peer coach, was perceived by participants as particularly valuable. Peer coaches tailored their guidance to individual questions and real-life case experiences shared by nurses, supporting reflection that was directly linked to everyday practice.

However, several limitations warrant consideration when interpreting these findings. The absence of formal member checking may have reduced credibility, and triangulation of behavioural change was limited by reliance on qualitative data only and by the absence of observational data or other independent indicators of behavioural change. The peer-coach/colleague context may have increased social desirability. Recruitment via a care manager and nurses’ participation within workplace training (although voluntary) may have introduced gatekeeping, self-selection and perceived obligation. In addition, focus group dynamics and caregiver presence during interviews with people with dementia may also have shaped responses. As with most qualitative feasibility studies, researcher involvement in data collection and interpretation may also have influenced the analysis, although reflexive discussions within the research team aimed to mitigate this risk.

The programme’s complexity and time demands contributed to informal caregiver burden and partial dropout, potentially introducing selection bias. In-person participation was constrained by staff shortages and scheduling difficulties, necessitating digital delivery. While this offered flexibility, it may have altered group dynamics and relational engagement. The lack of a structured preintervention inventory limits causal inference, and the long-term durability of observed behavioural change remains unknown. While we monitored programme exposure (eg, reflective diary completion), assessing fidelity of enactment—how consistently nurses applied intervention principles in routine home-care encounters—remains challenging in real-world practice without direct observation.

As this was a feasibility study with a limited sample size and duration, findings should be interpreted as exploratory. Finally, as the study was conducted within a single national context and care system—characterised by specific organisational conditions, workforce constraints and cultural understandings of self-direction—the generalisability of findings to other settings may be limited. Together, these limitations highlight the need for larger, comparative and observational studies to confirm programme’s effectiveness and sustainability.

### Mechanisms of change

Behavioural change among participants appeared to be driven by structured reflection, experiential learning and a gradual mindset shift. Nurses reported becoming more aware of inconsistencies between their values and routines, a finding consistent with Grol *et al*,^43^ who emphasise the role of reflection in sustaining behaviour change. The iterative, longitudinal format of SOCAV-HC enabled participants to build confidence through incremental progress. This resonates with theories from Mareš^44^ and Simons and Ruijters,^45^ on the integration of reflection and experience as mechanisms for professional development. However, consistent with prior literature, participants initially struggled to translate abstract care principles like self-direction into concrete action, particularly under time pressure. Resistance to change was common at first, but gradually diminished through practice, reflection and dialogue.

### Implementation challenges and sustainability strategies in home care

Implementing SOCAV-HC in home care revealed challenges, particularly role ambiguity between nurses and informal caregivers, which often led to miscommunication. This is consistent with prior research,^46^ which highlights how multiple actors in the home setting can blur responsibilities and complicate shared decision-making. Structured meetings and training helped mitigate these issues by fostering mutual understanding.

Nevertheless, translating the concept of self-direction into daily routines remained difficult. Although informal caregivers and nurses endorsed the idea in principle, they often lacked strategies to enact it. This finding mirrors earlier studies indicating that abstract care principles such as autonomy require shared frameworks and practical tools to guide successful implementation.^47^ Within SOCAV-HC, reflective coaching and the use of diaries helped bridge this gap by linking principles to concrete experiences in practice. Over time, positive experiences—such as improved communication and greater self-direction for people with dementia—reinforced new behaviours. The sustainability of these changes, however, depends on more than individual effort. Embedding self-direction in daily care requires consistent support at multiple, interconnected levels: research and programme development, organisational vision and infrastructure, professional teams and individual care practice.

To guide future implementation and ensure long-term integration, we propose a set of multi-level strategies (see box 1). These are informed by experiences during the feasibility phase and are intended to support the ongoing adaptation and contextualisation of SOCAV-HC within dementia home care.

Box 1 **Multilevel strategies to support the sustainable implementation of SOCAV-Home Care**

The sustainability of SOCAV-Home Care depends on embedding self-direction across four levels:

- Research and programme development: Co-create practical tools and share learning across settings.
- Organisational vision: Integrate self-direction into policy, training and quality frameworks.
- Professional teams: Use brief reflections and team dialogues to make self-direction part of everyday care.
- Individual care practice: Maintain self-direction–supportive care through ongoing reflection, shared learning and attention to small daily choices that strengthen trust and continuity.

Sustainability arises when self-direction becomes an organisational mindset rather than a discrete intervention.

### Unanswered questions and directions for future research

Reflective diaries revealed a shift toward more integrated, person-centred care that encompassed not only physical needs, but also psychosocial and organisational dimensions, contributing to a more relational model of dementia care. To build on this foundation, several improvements are suggested: enhanced scheduling flexibility, differentiated training tracks, clearer conceptual frameworks, expanded digital options and incorporating professional boundaries as a topic in coaching. These insights point towards reframing self-direction not as a static individual capacity, but as an evolving and context-sensitive process embedded in daily care relationships. The sustainability strategies outlined in box 1 align with these findings and offer a practical foundation for further implementation efforts; a more detailed version of box 1 is provided in online supplemental file 3. Future studies should investigate the sustainability of these changes, their transferability to other settings and long-term impacts on quality of life for people with dementia and their caregivers.

### Conclusions

SOCAV-HC shows strong potential to enhance person-centred dementia care at home by strengthening relational care practices and embedding self-direction into everyday routines. Its reflective and collaborative format provides a foundation for more psychosocially responsive care. However, conclusions regarding effectiveness and long-term impact still require further evaluation using independent indicators of behavioural change and longer follow-up. In light of the feasibility design and scope of the study, the findings should therefore be regarded as promising but not yet conclusive. To enable sustainable implementation, future efforts should focus on reducing complexity, clarifying key concepts and increasing flexibility.

## Supplementary material

10.1136/bmjopen-2025-105939 online supplemental file 1

10.1136/bmjopen-2025-105939 online supplemental file 2

10.1136/bmjopen-2025-105939 online supplemental file 3

## Data Availability

Data are available on reasonable request. All data relevant to the study are included in the article or uploaded as supplementary information.

## References

[1] Lethin C, Leino-Kilpi H, Bleijlevens MH (2020). Predicting caregiver burden in informal caregivers caring for persons with dementia living at home - A follow-up cohort study. Dementia (London).

[2] Bunn F, Goodman C, Sworn K (2012). Psychosocial factors that shape patient and carer experiences of dementia diagnosis and treatment: a systematic review of qualitative studies. PLoS Med.

[3] van Corven C, Bielderman A, Wijnen M (2022). Promoting empowerment for people living with dementia in nursing homes: Development and feasibility evaluation of an empowerment program. Dementia (London).

[4] Bjørkløf GH, Helvik A-S, Ibsen TL (2019). Balancing the struggle to live with dementia: a systematic meta-synthesis of coping. BMC Geriatr.

[5] Nimmons D, Manthorpe J, West E (2023). Views of people living with dementia and their carers on their present and future: a qualitative study. BMC Palliat Care.

[6] Groenewoud H, Senhaji H, van ’t Leven N (2017). Barriers and facilitators to improve the social network of home‐dwelling people living with dementia and their family caregivers. Alzheimer's & Dementia.

[7] van der Roest HG, Meiland FJM, Comijs HC (2009). What do community-dwelling people with dementia need? A survey of those who are known to care and welfare services. Int Psychogeriatr.

[8] Keady J, Nolan M (2020). Person-and relationship-centred dementia care.

[9] Kitwood T (1997). Dementia Reconsidered: The Person Comes First.

[10] van Corven CTM, Bielderman A, Wijnen M (2021). Defining empowerment for older people living with dementia from multiple perspectives: A qualitative study. Int J Nurs Stud.

[11] Meinema T (2019). What works to stimulate self-direction.

[12] Fetherstonhaugh D, Tarzia L, Nay R (2013). Being central to decision making means I am still here!: the essence of decision making for people with dementia. J Aging Stud.

[13] McCormack B, McCance T, Bulley C (2021). Fundamentals of Person-Centred Healthcare Practice.

[14] Kang B, Scales K, McConnell ES (2020). Nursing home residents’ perspectives on their social relationships. J Clin Nurs.

[15] Argyle E, Kelly T, Gladman J (2017). The effective ingredients of social support at home for people with dementia: a literature review. *JICA*.

[16] Graff MJL, Vernooij-Dassen MJM, Thijssen M (2006). Community based occupational therapy for patients with dementia and their care givers: randomised controlled trial. BMJ.

[17] Graff M, Melick M, Thijssen M (2010). Occupational therapy for dementia patients and their primary caregivers.

[18] Kielhofner G (2009). Conceptual Foundations of Occupational Therapy Practice.

[19] Townsend E, Galipeault JP, Gliddon K (2003). Reflections on Power and Justice in Enabling Occupation. *Can J Occup Ther*.

[20] Metzelthin SF, Rostgaard T, Parsons M (2022). Development of an internationally accepted definition of reablement: a Delphi study. Ageing Soc.

[21] Döpp C, Heide I, Hein M (2017). Figures from the National Dementia Care and Support Register For.

[22] Graff MJL, Vernooij-Dassen MJM, Zajec J (2006). How can occupational therapy improve the daily performance and communication of an older patient with dementia and his primary caregiver?. Dementia (London).

[23] Roets-Merken LM, Zuidema SU, Vernooij-Dassen MJFJ (2018). Effectiveness of a nurse-supported self-management programme for dual sensory impaired older adults in long-term care: a cluster randomised controlled trial. BMJ Open.

[24] Roets-Merken LM, Vernooij-Dassen MJFJ, Zuidema SU (2016). Evaluation of nurses’ changing perceptions when trained to implement a self-management programme for dual sensory impaired older adults in long-term care: a qualitative study. BMJ Open.

[25] Das P, Douma G, Donkers H (2026). SOCAV, a nurse-led program promoting self-direction in nursing homes: a longitudinal mixed-methods pilot study. BMC Geriatr.

[26] Craig P, Dieppe P, Macintyre S (2013). Developing and evaluating complex interventions: The new Medical Research Council guidance. Int J Nurs Stud.

[27] Skivington K, Matthews L, Simpson SA (2021). A new framework for developing and evaluating complex interventions: update of Medical Research Council guidance. BMJ.

[28] Bowen DJ, Kreuter M, Spring B (2009). How we design feasibility studies. Am J Prev Med.

[29] de Vaate M-B, van der Weele G, Schep-Akkerman A (2020). Herziene NHG-Standaard Dementie. Huisarts Wet.

[30] Sheikh JI, Yesavage JA (2014). Geriatric Depression Scale (GDS): recent evidence and development of a shorter version. Clinical Gerontology: Routledge.

[31] Graff MJL, Vernooij-Dassen MJM, Thijssen M (2007). Effects of community occupational therapy on quality of life, mood, and health status in dementia patients and their caregivers: a randomized controlled trial. J Gerontol A Biol Sci Med Sci.

[32] Staniszewska S, Brett J, Simera I (2017). GRIPP2 reporting checklists: tools to improve reporting of patient and public involvement in research. BMJ.

[33] Law M, Baptiste S, McColl M (1990). The Canadian occupational performance measure: an outcome measure for occupational therapy. Can J Occup Ther.

[34] Tuntland H, Aaslund MK, Langeland E (2016). Psychometric properties of the Canadian Occupational Performance Measure in home-dwelling older adults. J Multidiscip Healthc.

[35] Brod M, Stewart AL, Sands L (1999). Conceptualization and measurement of quality of life in dementia: the dementia quality of life instrument (DQoL). Gerontologist.

[36] Droës R (1993). Dutch Translation of the Cornell-Scale for Depression in Dementia.

[37] Kaufer DI, Cummings JL, Ketchel P (2000). Validation of the NPI-Q, a brief clinical form of the Neuropsychiatric Inventory. J Neuropsychiatry Clin Neurosci.

[38] Lutomski JE, Baars MAE, Schalk BWM (2013). The development of the Older Persons and Informal Caregivers Survey Minimum DataSet (TOPICS-MDS): a large-scale data sharing initiative. PLoS One.

[39] Corbin J, Strauss A (2014). Basics of qualitative research: techniques and procedures for developing grounded theory: Sage publications.

[40] Soratto J, Pires D de, Friese S (2020). Thematic content analysis using ATLAS.ti software: Potentialities for researchs in health. Rev Bras Enferm.

[41] SPSS I. IBM Corp (2017). Released, Statistics for Windows, Version 25.0. Armonk.

[42] Tong A, Sainsbury P, Craig J (2007). Consolidated criteria for reporting qualitative research (COREQ): a 32-item checklist for interviews and focus groups. Int J Qual Health Care.

[43] Grol R, Wensing M, Eccles M (2013). Improving Patient Care: The Implementation of Change in Health Care.

[44] Mareš J (2018). Resistance of health personnel to changes in healthcare. Kontakt.

[45] Simons P-J, Ruijters MC (2014). International handbook of research in professional and practice-based learning.

[46] Fiscella K, McDaniel SH (2018). The complexity, diversity, and science of primary care teams. Am Psychol.

[47] Vluggen S, Metzelthin S, Lima Passos V (2022). Effect, economic and process-evaluation of a generic function focused care program for long-term care; study protocol of a multicenter cluster-randomized trial. BMC Nurs.

