# Supplementary material for: SOCAV: a nurse-led support programme for self-direction in people with dementia receiving home care, involving informal caregivers – a feasibility study with process evaluation in the Netherlands
Source: BMJ Open. 2026 Mar 18;16(3):e105939. doi: 10.1136/bmjopen-2025-105939 (PMC13007093; doi:10.1136/bmjopen-2025-105939)
Supplement: online supplemental file 1 [file bmjopen-16-3-s001.docx]

**Supplementary File 1**

**Interview guides**

**Overview:**

**1. Development phase**

*Interview guide for focus group informal caregivers (5 months)*

*Interview guide for focus group nurses (5 months)*

*Guide for interview with peer coaches (5 months)*

**2. Feasibility phase**

*Interview guide for focus groups informal caregivers – Baseline*

*Interview guide for focus group nurses (midpoint)*

*Interview guide: Baseline outcome measurements feasibility phase*

**1. Development phase**

*Interview guide for focus group informal caregivers (5 months)*

Purpose: To better understand your experiences with SOCAV, your interactions with staff, and your views on self-direction and autonomy in caring for family members. You are experts by experience, and we would like to learn from you.

Note: We would like to use the insights from this conversation in the next phase. Do you agree that relevant points may be shared anonymously in a future meeting?

1. What was your experience during the SOCAV period?

a. Were there any factors that disrupted the process? If so, which?

2. What did you think about how Avoord explained or communicated SOCAV?

a. What did you think was good/less good? What did you miss?

3. Has SOCAV influenced how you support your family member?

Or: How do you view SOCAV? Have things changed for you, or for/with your family member?

a. Do you notice any difference in your family member’s reactions or behaviour?

b. Has SOCAV resulted in your family member making more decisions for themselves? If so, could you give an example? In what ways do you provide support?

c. How did you experience the coaching conversations?

4. During the previous focus group in June, I asked how you would describe self-direction/autonomy. How do you view self-direction/autonomy now?

5. Do you feel sufficiently equipped to apply person-centred care (SOCAV)? Why do you think that?

a. What knowledge or skills do you think you still need, or felt were missing?

6. You participated in a pilot—what should definitely stay the same, and what could be done differently (with regard to the next phase)? What should be retained?

- Has it been burdensome?

- What support do you need to be able to continue applying SOCAV in the future?

*Interview guide for focus group nurses (5 months)*

Aim: To gain insight into your experiences of working with SOCAV, your contact with clients, and your views on the concept of self-direction/autonomy. You are now experts by experience, and we would like to learn from you.

1. How have you experienced working with SOCAV? And the coaching?

2. Have there been any factors that disrupted the learning process? What were you able or unable to do? (e.g., time pressure, etc.)

3. Are there things you have started doing differently in your work with people with dementia?

4. Do you notice any difference in the client’s response? What do you pay attention to?

5. During the previous focus group in June, I asked how you would describe self-direction/autonomy. How do you view self-direction/autonomy now?

6. You participated in this pilot—what should definitely stay the same, and what could be done differently (with regard to the next phase)? What should be retained?

*Guide for interview with peer coaches (5 months)*

Aim: To gain insight into the experiences of the peer coaches and to exchange ideas on how the SOCAV training and intervention might be improved.

1. What was your experience during the SOCAV period?

a. In your view, what were the key success factors?

b. What are points we should look at critically?

c. What did you observe regarding need/demand, and whether SOCAV was a good fit?

d. Do you think there is a need—and do participants have an idea of what they are getting into?

e. Also regarding the training: what were the success factors and critical factors?

2. Ralf, how did the recruitment/approach process go? You called people—how did that go? What did you encounter?

a. What were you satisfied with? What were the key points?

b. How were you able to generate enthusiasm while still being realistic?

c. Do you think people could oversee what it would involve—what was their attitude?

3. It has become clear that scheduling the individual coaching sessions is difficult, and possibly also starting the home-based intervention with the informal carer and the person with dementia. How can we support this?

4. A new coach will be joining (if all goes as planned). How can we best support/prepare him or her?

a. Peer consultation/intervision between the coaches?

5. Do you have any further ideas we should consider?

**2. Feasibility phase**

*Interview guide for focus groups informal caregivers – Baseline*

Aim: To gain insight into how informal carers support their partner, parent, neighbour, etc., and to what extent they are aware of their own way of providing support.

5. If you think about the way you support your partner/parent/neighbour with dementia, what are you proud of? (Optional: specify “in the past two weeks”.)

a. Why did you do it that way?

b. Did it suit him or her? How did you arrive at that approach?

i. How did you know?

- Consider bringing in interaction by asking whether others recognise this, or whether there are surprising points that lead to new insights.

6. To what extent do you experience good coordination with home care services regarding the approach or the way care is delivered, in a way that fits your partner/parent/neighbour?

a. If yes, how did this come about?

b. Where could it be improved?

7. When I talk about self-direction/autonomy, what comes to mind for you?

- At the end, summarise to ensure clarity and a shared understanding of the concept.

8. Do you have the sense that your partner/mother/neighbour still experiences self-direction/autonomy?

- Possible examples: being able to decide what he/she will do that day; choosing to go somewhere; choosing to take part in an activity.

- How? Why or why not?

- Do you think it is important?

- Can you give an example of a specific moment when you try to encourage self-direction/autonomy?

- Do you receive support with this (for both of you)? Would you consider that important?

Switch off recorder.

Close the session and request details for member checking.

*Interview guide for focus group nurses (midpoint)*

Preliminary notes

Please mention informed consent.

Purpose of the discussion

The aim of this discussion is to gain a better understanding of how SOCAV fits within the teams, and to identify any indications that elements may need to be adjusted.

We will reflect on:

The recent period of SOCAV training and coaching (experiences, intensity, successes, barriers, etc.).

The kitchen-table conversations: who has experienced these already, and what was that like? (shift from focusing on one’s own learning process to focusing on the client and informal carer—how is that?).

Zooming in on SOCAV and the learning process, including your questions about “working from the SOCAV approach”.

In short: your practical experiences—how is it going now, what opportunities and challenges do you see, what support would you like, and what are your wishes and needs for the future?

The focus group will take approximately 60 minutes.

Questions

General: how is SOCAV going?

First, we are interested in a brief overview of how things are going.

1. Could you tell us how things are going in your team at the moment? (general impression)

a. Is SOCAV still “alive”? How do you notice this?

b. If you look at yourself and your work with SOCAV, what score would you give yourself?

2. Have there been any factors that disrupted the learning process? What were you able or unable to do? (e.g., COVID-19, time pressure, etc.)

Looking back on the past period

Training and coaching

1. What was your experience during the SOCAV period?

a. It continued online—how did you find that?

2. How do you experience the individual sessions?

a. How often do they take place? What do you think of the frequency? Your experience/feelings about it?

b. Looking back over the period: what have the individual sessions given you personally?

c. Could you give an example—e.g., a question you brought in, or a situation?

3. The group training has ended. How can you continue learning as a group in the coming period?

4. Do you feel you have received enough practical tools to continue applying SOCAV?

a. What was helpful for you?

b. What do you still miss?

Kitchen-table conversations

5. Are you taking part in the kitchen-table conversations? How do you experience these?

Working from the SOCAV approach

1. How do you experience working from SOCAV?

a. Is it going reasonably well? (examples/experiences)

b. To what extent do clients themselves already determine what is done? What is your feeling about that? Could you give a practical example? Do you find this helpful or difficult, and why?

2. In what ways do you notice you have started doing things differently in your work with people with dementia?

a. Could you give an example? How did that feel?

b. Do you notice any difference in the client’s response? What do you pay attention to?

3. How do you now view client autonomy/self-direction?

a. Do you apply SOCAV with people with dementia at home, or more broadly?

Looking ahead: sustaining SOCAV

1. How would you like to continue with SOCAV, and what do you need for that?

Relationship with management

2. As a team (or as an individual), do you need anything from management and/or your team leader?

- Do you feel there is sufficient involvement and support for SOCAV?

- Has SOCAV been discussed again since the start—for example during team meetings? In what way?

- How would you like SOCAV to come up in the future?

- During regular team meetings, or separately?

Thank you very much for your input!

*Interview guide: Baseline outcome measurements feasibility phase*

Administration of measurement instruments:

Administer primary measurement instruments/questionnaires.

Secondary measurement instruments/questionnaires are optional (if participants have sufficient capacity, administer immediately afterwards; or leave the questionnaire with a request to complete it; or administer by telephone 1–2 days after the appointment).

Add two questions: perceived burden and how confronting the assessment was, each on a 1–10 scale.

Plan a 2-hour appointment for the baseline measurements.

Baseline (T0): primary measurement instruments

1. COPM (30 minutes) administered with the client, using the OPHI-II to introduce the COPM (the informal carer may be present, but should not participate in the conversation). Use a 10 cm line with smiley faces instead of numbers for scoring.

2. Administer DQoL with the client (without the informal carer present).

3. Administer COPM with the informal carer, asking about the carer’s perspective on the client’s goals.

4. Administer CES-D with the informal carer.

5. Conduct an ethnographic interview and an (individual) COPM with the informal carer.

6. Communicate COPM outcomes, so that SOCAV training can be tailored to these goals. Inform participants that information will be shared.

Materials needed (primary instruments)

OPHI form (Edomah).

Ethnographic interview form (Edomah).

COPM form for the client.

COPM scoring form, with extra space for the informal carer’s perspective/notes.

10 cm strip with smiley faces for client scoring.

DQoL form.

CES-D form.

Baseline (T0): secondary measurement instruments (optional / to leave behind if needed)

TOPICS-short (client).

CORNELL (informal carer).

NPI-Q (informal carer).

TOPICS (informal carer).

Materials needed (secondary instruments)

TOPICS-short form (client).

CORNELL form (informal carer).

NPI-Q form (informal carer).

TOPICS-MDS form (informal carer).

Introduction script: client

Thank you for taking part in the SOCAV at home study. Today I would like to get to know you better, and I will therefore conduct an interview and administer questionnaires with you. This appointment marks the start of the study and will take approximately 2 hours. During this time, I will speak both with you and with your informal carer.

Please let me know if you would like a break, or if you do not want to continue with the interview or questionnaires.

Do you have any questions about this study?

Today we will start with a conversation about how you used to organise your life, which things were important to you, and which roles you had. After that, we will look at your life at the moment: which activities you enjoy, which you would like to do, and how you feel these activities are going. Your informal carer may be present, but I would like to ask you to answer the questions yourself.

Start OPHI-II and COPM with the client (integrate the explanation of the COPM into the conversation after the OPHI introduction).

Now I would like to continue by completing a questionnaire. These questions are about your quality of life, that is, how you experience your life at the moment. I would like to administer this questionnaire without your informal carer being able to follow the conversation. Later, I would like to ask similar questions to your informal carer.

Start DQoL.

Optional (choose one option):

I have one more questionnaire that I would like to complete with you.

I have one more questionnaire—may I call you tomorrow or the day after tomorrow to complete it together by phone?

I have one more questionnaire—would you be willing to complete it yourself and return it to me by post? A stamped addressed envelope is included.

Start TOPICS-short.

Introduction script: informal caregiver

Now I would also like to administer a number of questionnaires with you. These relate both to the answers your family member has just given and to your own situation and experiences.

Please let me know if you would like a break, or if you do not want to continue with this interview and the questionnaires.

Do you have any questions about the study at this moment?

Your family member has just indicated which activities are important to them and how satisfied they are with these activities. I would like to discuss the answers given with you. Could you share your thoughts on the answers? What is your perspective?

Start COPM (perspective).

The next part is a questionnaire that I would like to administer with you. This questionnaire relates to yourself. You may complete it yourself, or we can do it together—whichever you prefer.

Start CES-D.

This questionnaire is about your family member; it includes questions about, among other things, mood and behaviour.

Start CORNELL (CSDD).

The next questionnaire is administered as a dialogue. I will go through the questions together with you. It is about the changes you see, or have seen, in your family member.

Start NPI-Q.

In addition, we are interested in your experiences as an informal carer. You can complete this questionnaire yourself now. You may also choose to do it at a later time and return the completed questionnaire to us, or we can schedule an appointment to complete it together by phone.

Start TOPICS.
