# Supplementary material for: SOCAV: a nurse-led support programme for self-direction in people with dementia receiving home care, involving informal caregivers – a feasibility study with process evaluation in the Netherlands
Source: BMJ Open. 2026 Mar 18;16(3):e105939. doi: 10.1136/bmjopen-2025-105939 (PMC13007093; doi:10.1136/bmjopen-2025-105939)
Supplement: online supplemental file 2 [file bmjopen-16-3-s002.docx]

**Supplementary File 2**

**Conversation Topics Table from the SOCAV-Home Care Study**

Categorization of topics addressed in conversations between nursing staff and individuals with dementia during home care, as noted in nurses’ diaries

**Note:** The following inventory categorizes topics addressed during conversations between nursing staff and individuals with dementia during home care. These conversations formed the foundation for reflections recorded in nurses’ diaries and were subsequently discussed in coaching sessions with the SOCAV peer coach. The topics are grouped into three categories: Care, Organization, and Socio-emotional, providing insights into the diverse issues that home care workers encounter daily while practicing the SOCAV program. While this categorization is not rigid or definitive, it offers a valuable overview of the variety of subjects addressed and illustrates the broad range of challenges and opportunities that nursing staff identify and address in their work.

### **Care:**

Includes topics related to daily care activities, such as bathing, dressing, medication management, assistance with feeding, and mobility assistance.

### **Organization:**

Covers planning care moments, coordinating tasks among caregivers, and handling logistics related to appointments with physicians, such as scheduling doctor visits or meal planning.

### **Socio-emotional:**

Focuses on social and emotional needs, including identifying loneliness, providing emotional support, and observing behavioral changes.

|  | **Discussion Topic** | **Observed/**  **Felt by** | | **Category** |
| --- | --- | --- | --- | --- |
|  | Laces in shoes | Person with dementia | | Care |
|  | Painful blister | Person with dementia | | Care |
|  | Aid for putting on support stockings independently | Person with dementia | | Care |
|  | Blow-drying hair after showering | Person with dementia | | Care |
|  | What would you like to drink | Home care nurse | | Care |
|  | Stoma care | Home care nurse | | Care |
|  | Going to the toilet independently | Person with dementia | | Care |
|  | Wanting to shower independently | Person with dementia | | Care |
|  | No longer wanting support stockings | Person with dementia | | Care |
|  | Care for a painful knee | Person with dementia | | Care |
|  | Applying lotion on the back | Person with dementia | | Care |
|  | Pain | Person with dementia | | Care |
|  | Difficulty taking medication | Person with dementia | | Care |
|  | Fall risk observed | Home care nurse | | Care |
|  | Fatigued after showering independently | Person with dementia | | Care |
|  | Fatigued due to independent ADL care | Person with dementia | | Care |
|  | Forgetting to take medication | Person with dementia | | Care |
|  | Scheduling eye drops | Person with dementia | | Care |
|  | Bed trapeze (overhead assistive device) | Person with dementia | | Care |
|  | Taking over all personal care | Person with dementia | | Care |
|  | Help with washing underwear independently | Person with dementia | | Care |
|  | Catheter leakage | Person with dementia | | Care |
|  | Fell (observed by home care nurse) | Home care nurse | | Care |
|  | Ergonomic bandaging | Home care nurse | | Care |
|  | Wanting to apply eye drops independently | Person with dementia | | Care |
|  | Applying eye drops independently | Person with dementia | | Care |
|  | Managing insulin independently | Person with dementia | | Care |
|  | Showering independently | Person with dementia | | Care |
|  | Help with showering | Person with dementia | | Care |
|  | No more care on the ground floor | Person with dementia | | Care |
|  | Treating a small wound | Person with dementia | | Care |
|  | Wound on the foot | Person with dementia | | Care |
|  | Forgotten how to put on support stockings | Person with dementia | | Care |
|  | Many clothes taken out of the closet | Home care nurse | | Care |
|  | Fall risk | Person with dementia | | Care |
|  | Urine in bed | Home care nurse | | Care |
|  | Do not put on support stockings in bed | Person with dementia | | Care |
|  | Putting on shoes by home care staff | Person with dementia | | Care |
|  | Pain | Person with dementia | | Care |
|  | Feeling unsafe while showering | Person with dementia | | Care |
|  | Applying lotion on lower legs | Person with dementia | | Care |
|  | Difficulty swallowing medication | Person with dementia | | Care |
|  | Prefer not to have foot cream | Person with dementia | | Care |
|  | Putting on stockings independently | Person with dementia | | Care |
|  | Tidying up clothes taken from the closet | Home care nurse | | Care |
|  | Choice of what and how much to eat | Person with dementia | | Care |
|  | Incontinence | Person with dementia | | Care |
|  | Aid for getting out of bed | Person with dementia | | Care |
|  | Help with dressing | Home care nurse | | Care |
|  | Washing hair | Person with dementia | | Care |
|  | Using a urinal | Person with dementia | | Care |
|  | Extra shower | Person with dementia | | Care |
|  | Showering or washing? | Home care nurse | | Care |
|  | Drying the shower | Person with dementia | | Care |
|  | Blood on bed sheets | Home care nurse | | Care |
|  | Different approach to ADL | Home care nurse | | Care |
|  | Regain strength | Home care nurse | | Care |
|  | Purchasing dd (?) | Person with dementia | | Organization |
|  | Finding a TV program | Person with dementia | | Organization |
|  | Knowing the time when a volunteer will arrive | Person with dementia | | Organization |
|  | Torn pyjama | Person with dementia | | Organization |
|  | Planning a doctor visit | Person with dementia | | Organization |
|  | Visiting family | Person with dementia | | Organization |
|  | Different meals | Person with dementia | | Organization |
|  | Adjustments in the apartment | Person with dementia | | Organization |
|  | What do you want after passing away? | Home care nurse | | Organization |
|  | Evaluation appointment with doctor | Home care nurse | | Organization |
|  | Unclear agreements with home care | Person with dementia and informal caregiver | | Organization |
|  | Wanting to visit a friend | Person with dementia | | Organization |
|  | Home care late for appointment | Person with dementia | | Organization |
|  | Stopping home care | Person with dementia | | Organization |
|  | Visiting a friend | Person with dementia | | Organization |
|  | Calling the GP | Person with dementia | | Organization |
|  | Wanting daycare for partner | Informal caregiver | | Organization |
|  | Going outside through the emergency exit | Person with dementia | | Organization |
|  | Cleaning up plastic waste | Person with dementia | | Organization |
|  | Partner wants to accompany to the GP | Person with dementia | | Organization |
|  | Feeling unsafe due to a broken window | Person with dementia | | Organization |
|  | More exercise and going outdoors | Person with dementia | | Organization |
|  | No clear information on when home care will arrive | Person with dementia | | Organization |
|  | Purchasing a drip glass for eyedrops | Person with dementia | | Organization |
|  | Temporarily needs 24-hour care | Home care nurse | | Organization |
|  | Home care arrived late | Person with dementia | | Organization |
|  | New mattress | Person with dementia | | Organization |
|  | Arranging meals independently | Person with dementia | | Organization |
|  | Sending a photo via social media | Person with dementia | | Organization |
|  | Showering at a different time, wants to watch press conference on TV | Person with dementia | | Organization |
|  | Putting on blue slippers when entering | Person with dementia | | Organization |
|  | No longer allowing a specific home care staff member in | Person with dementia | | Organization |
|  | Appointment with GP regarding euthanasia | Person with dementia | | Organization |
|  | Cognitive assessment for partner | Informal caregiver | | Organization |
|  | Care during hollidays | Person with dementia | Organization | |
|  | Arranging a taxi independently | Person with dementia | Organization | |
|  | Wound care during hollidays | Person with dementia | Organization | |
|  | Wants to watch football during care time | Person with dementia | Organization | |
|  | Laundry in the dryer | Person with dementia | Organization | |
|  | Time for guidance conversation | Home care nurse | Organization | |
|  | Planning a trip | Person with dementia | Organization | |
|  | Pedicure too expensive | Person with dementia | Organization | |
|  | Being on time for the wheelchair taxi | Person with dementia | Organization | |
|  | Informal caregiver wants to continue living with partner | Mz | Organization | |
|  | Organizing the folder clearly | Person with dementia | Organization | |
|  | Meals delivered by neighbors | Person with dementia | Organization | |
|  | Knee brace delivered too late | Person with dementia | Organization | |
|  | Caregiver did not show up | Person with dementia | Organization | |
|  | Talking to the GP | Person with dementia | Organization | |
|  | How does home care enter the house? | Person with dementia | Organization | |
|  | Filling in forms | Person with dementia | Organization | |
|  | Food delivered too late | Person with dementia | Organization | |
|  | Purchasing a drip glass for eyedrops | Person with dementia | Organization | |
|  | Neighbor should not interfere | Person with dementia | Organization | |
|  | Calling the GP | Person with dementia | Organization | |
|  | Evening care at an earlier time | Person with dementia | Organization | |
|  | Showering at a different time | Person with dementia | Organization | |
|  | Rescheduling a home care appointment | Person with dementia | Organization | |
|  | Making an appointment with the dentist | Person with dementia | Organization | |
|  | Walk with partner | Person with dementia | Organization | |
|  | Going outside | Person with dementia | Organization | |
|  | Going out with the mobility scooter | Person with dementia | Organization | |
|  | Walking therapy, whether or not to walk now | Home care nurse | Organization | |
|  | Doubts about medication | Person with dementia | Social-emotional | |
|  | How was it in the hospital? | Home care nurse | Social-emotional | |
|  | Panicking about which home care staff will come | Person with dementia | Social-emotional | |
|  | Emotional, need to talk | Person with dementia | Social-emotional | |
|  | Feeling lonely | Person with dementia | Social-emotional | |
|  | Wanting to be among people | Person with dementia | Social-emotional | |
|  | Wanting to go outside, wanting to leave | Person with dementia | Social-emotional | |
|  | Seeking contact with home care staff | Home care nurse | Social-emotional | |
|  | Appearing sad | Home care nurse | Social-emotional | |
|  | Issues with a specific home care staff member | Person with dementia and informal caregiver | Social-emotional | |
|  | Seeing hallucinations (children) | Person with dementia | Social-emotional | |
|  | Confused, calling for help | Person with dementia | Social-emotional | |
|  | Desperate, unable to express what is wrong | Person with dementia | Social-emotional | |
|  | Accidentally activating the alarm | Person with dementia | Social-emotional | |
|  | Sparing and helping the partner | Person with dementia | Social-emotional | |
|  | Forgetting the name of the hometown | Person with dementia | Social-emotional | |
|  | Difficulty reading | Person with dementia | Social-emotional | |
|  | Wanting more social contacts | Person with dementia | Social-emotional | |
|  | Feeling tired of life | Person with dementia | Social-emotional | |
|  | Annoyed by frequent calls from a friend with problems | Person with dementia | Social-emotional | |
|  | Wanting children to visit more often | Person with dementia | Social-emotional | |
|  | Feeling lonely | Person with dementia | Social-emotional | |
|  | Family issues | Person with dementia | Social-emotional | |
|  | Feeling lonely and in poor condition | Person with dementia | Social-emotional | |
|  | Appears tired | Home care nurse | Social-emotional | |
|  | Does not want to go to bed | Person with dementia | Social-emotional | |
|  | Wants to be less dependent | Person with dementia | Social-emotional | |
|  | Does not want a COVID-19 vaccination | Person with dementia | Social-emotional | |
|  | Confusion | Home care nurse | Social-emotional | |
|  | Talking about deceased partner | Person with dementia | Social-emotional | |
