# Supplementary material for: SOCAV: a nurse-led support programme for self-direction in people with dementia receiving home care, involving informal caregivers – a feasibility study with process evaluation in the Netherlands
Source: BMJ Open. 2026 Mar 18;16(3):e105939. doi: 10.1136/bmjopen-2025-105939 (PMC13007093; doi:10.1136/bmjopen-2025-105939)
Supplement: online supplemental file 3 [file bmjopen-16-3-s003.docx]

## Supplementary File 3

## Box 1. Multi-level strategies to support the sustainable implementation of SOCAV-Home Care in dementia care.

Although care providers and organisations broadly agree on the importance of self-direction, many struggle to recognise and support it in daily care. The sustainability of SOCAV-Home Care depends on embedding this principle across four interconnected levels: research and programme development, organisational vision and infrastructure, professional teams, and individual care practice.

### 1. Research and Programme Development

- Develop clear and adaptable materials that show what self-direction looks like in practice.
- Co-create tools with professionals, caregivers, and people with dementia to ensure relevance and shared ownership.
- Use feedback loops to monitor how SOCAV is applied and adapted across contexts.
- Share learning across settings through networks that exchange practical experiences.

### 2. Organisational Vision and Infrastructure

- Adopt self-direction as a core organisational value before introducing SOCAV; the programme should grow from this shared vision.
- Integrate SOCAV principles into policy, training, and quality frameworks to ensure long-term continuity.
- Support reflection and coordination by designating facilitators and providing practical resources.
- Review progress regularly to keep self-direction visible in everyday decision-making.

### 3. Professional Teams

- Reflect on care moments where self-direction became visible and discuss what enabled it.
- Encourage open dialogue between professionals and caregivers to align understanding.
- Use simple tools—for example, short prompts or visual summaries—to help teams share insights and sustain learning.
- Treat self-direction as part of normal care, not as an additional task.

### 4. Individual Care Practice

- Start small: involve people with dementia in familiar daily choices to strengthen self-direction.
- Communicate interactively: nurses and caregivers should engage verbally and non-verbally to elicit preferences and co-create understanding.
- Reflect briefly on successful moments and note them to build awareness and skill.
- Value small shifts in self-direction as signs of growing trust and relational connection.

### Essence

Sustainability is achieved when self-direction is not treated as a separate intervention but as an enduring mindset – anchored in organisational vision, shared through team reflection, and enacted in every care interaction.
