# Supplementary material for: SOCAV: a nurse-led support programme for self-direction in people with dementia receiving home care, involving informal caregivers – a feasibility study with process evaluation in the Netherlands
Source: BMJ Open. 2026 Mar 18;16(3):e105939. doi: 10.1136/bmjopen-2025-105939 (PMC13007093; doi:10.1136/bmjopen-2025-105939)
Supplement: Supplementary file 4 [file bmjopen-2025-105939.reviewer_comments.pdf]

PEER REVIEW HISTORY

BMJ Open publishes all reviews undertaken for accepted manuscripts. Reviewers are asked to complete a checklist review form and are provided with free text boxes to elaborate on their assessment. These free text comments are reproduced below.

ARTICLE DETAILS

Title (Provisional)

SOCAV: a nurse-led support programme for self-direction in people with dementia receiving home care, including informal caregivers – a feasibility study with process evaluation in the Netherlands

Authors

Das, Phébe; Douma, Gerbrich; Donkers, Hanneke; Roets-Merken, Lieve; Graff, Maud

---

VERSION 1 - REVIEW

**Reviewer**	**1**
**Name**	**Stephan, Astrid**
**Affiliation**	**Uniklinik RWTH Aachen, Department of Nursing Science**
**Date**	**21-Jul-2025**
**COI**	**None**

This manuscript addresses an important and timely topic and presents a valuable contribution to the field. The manuscript is generally well written and provides interesting insights into the development of the intervention. However, several aspects of the methodology and reporting require clarification and more detailed description to enhance transparency and reproducibility.

Abstract Section

The methods employed in the study are not clearly described in the abstract. Specifically, the data collection methods are not presented. The authors only refer to the phases of the MRC Framework, which, in my opinion, is insufficient. For scientific transparency and comprehensibility, the abstract should briefly specify the concrete methods of data collection applied in the study.

Introduction Section

The introduction is well written and provides a solid foundation for the manuscript. However, it would benefit from a clearer elaboration of the international context and the

significance of the intervention. Strengthening these aspects would help underline the broader relevance and potential impact of the study beyond the immediate setting.

Please provide a more detailed explanation of how public involvement was implemented in the study. For example, it remains unclear whether this involvement took the form of a workshop, interviews, or if feedback was collected solely in written form. Clarifying these aspects would enhance the transparency and reproducibility of the methods.

Some important details are mentioned only at the end of the methods section, such as who conducted the interviews and who exactly comprised the research team. I initially thought this information was missing and almost overlooked it. Additionally, the terms mid-point and end-point are only explained at the end of the section. It would improve clarity and readability if such information were provided directly when first mentioned, for example by including brief explanations in parentheses.

The description of the outcome measures and the timing of their assessment (page 12) is not sufficiently clear. Presenting this information in a more structured way—for example, in the form of a table—could improve transparency and make it easier for readers to understand when and how each measure was collected.

On page 11, the term focus group interview is used once, whereas focus group is consistently used throughout the rest of the manuscript. For consistency and clarity, the terminology should be standardized. The term focus group is appropriate and should be used uniformly.

Please provide the exact duration of the focus groups (page 12).

The sentence "Interview guides were semi-structured and iteratively refined for relevance" (page 12, line 277) is unclear. Please elaborate on this process. Did you revise the guides after each interview or after completing a set of interviews? Who was involved in the refinement process, and based on which criteria were adjustments made?

Please clarify the format of the diaries used in the study—were they paper-based, online, or both? Additionally, it would be helpful to explain whether participants received specific guidelines or a predefined structure for the diary entries. What exactly were participants instructed to record?

Please specify the role and training of the coaches involved in the study. It remains unclear what their specific responsibilities were and what kind of preparation or qualifications they had.

On page 13, you state that you used content analysis. However, in English-language research, this term is often associated with quantitative content analysis. Please clarify whether you conducted a qualitative content analysis, and specify which approach or framework you followed.

On page 14, I suggest revising the section heading “Development phase: needs and perspectives on self-direction” to better reflect the content of this phase. Since the intervention was also modified during this phase, it would be clearer to include this aspect in the title. For example, you could adjust the heading to:

“Development phase: needs, perspectives on self-direction, and intervention adaptation.”

Overall, the results are clearly presented and engaging to read. The manuscript provides a comprehensive and well-structured account of the intervention development process, allowing readers to gain a detailed understanding of how the intervention was shaped. The discussion is appropriate and well-structured. It adequately reflects the study findings and places them in a meaningful context.

The manuscript would benefit from the inclusion of a figure summarizing the development process, the respective data collection points, and endpoints. Such a visual representation would enhance the clarity of the reporting and help readers to better grasp the sequence and structure of the study.

**Reviewer**	**2**
**Name**	**Woodward, Michael**
**Affiliation**	**Austin Health, Aged Care**
**Date**	**11-Dec-2025**
**COI**	**None**

This very important transfer of patient-centered care to residential care has been adequately presented, in this small 2-stage study.

The real question is sustainability- it would be good if you could provide some practical recommendations on how this could be achieved. Rather than just suggesting additional research, can you suggest ways this approach could be sustained, pending additional research?

VERSION 1 - AUTHOR RESPONSE

Reviewer 1

We thank the reviewer for the detailed and constructive feedback on the Methods. In response to comments 3–11, we rewrote and reorganised the Data Collection and Data Analysis sections to integrate all requested clarifications coherently within the main text. In addition, we added a dedicated section on programme implementation and delivery.

1. The abstract lacks information on data collection methods.
Response: The abstract now specifies both qualitative and quantitative methods, including

instruments used and qualitative content analysis.
(Page 2, lines 53–61)

2. The international context is underdeveloped.
Response: A new paragraph in the Introduction elaborates on the international relevance of self-direction in dementia care and the SOCAV approach.
(Page 6, lines 159–169)

3. Clarify how public involvement was implemented.
Response: A Patient and Public Involvement paragraph has been added, describing how and when input was gathered, based on the GRIPP2-SF checklist.
(Page 10, lines 291–304)

4. Key methodological details appear late in the Methods section.
Response: Key details such as interviewer roles, team composition, and terminology (e.g. “midpoint”) are now introduced earlier.
(Page 8, lines 230-231; page 10-11, data collection)

5. Outcome measures and timing are unclear.
Response: A new Table 1 has been added to present outcome measures, respondents, and timing across study phases. (Pages 12-13, Table 1)

6. Inconsistent terminology: “focus group interview” vs “focus group.”
Response: Terminology has been standardised to “focus group” throughout.

7. Please include the exact duration of the focus groups.
Response: Duration details have been added.
(Page 11, lines 337–338)

8. Elaborate on how interview guides were refined.
Response: Additional detail has been added on when, how, and by whom guides were refined.
(Page 11, lines 334-335)

9. Clarify diary format and instructions.
Response: The Implementation and Delivery section now clarifies that both paper-based and digital diaries were used and provides a general description of the prompts and instructions given to participants. (Pages 9–10, lines 275-288)

10. Specify the coaches’ role and training.
Response: The peer coaches’ role (guiding reflection and diary use) and training (five sessions over four months, including ongoing support) have been described in more detail.
(Page 9, lines 266–274)

11. Clarify “content analysis” — qualitative and framework.
We thank the reviewer for noting the potential ambiguity of “content analysis.” We have revised the manuscript to specify that we used qualitative content analysis guided by Bowen’s feasibility framework, complemented by a constant comparative approach

informed by Corbin and Strauss's analytic procedures, thereby clarifying both the qualitative nature and the framework-informed approach.

(Page 2, abstract, line 59; page 13, lines 373- 381)

12. Revise section heading on page 14 to include intervention adaptation.
Response: The heading has been revised to reflect the exploration of needs and intervention adaptation.
(Page 15, line 411)

13. A figure could help clarify development and data collection.
Response: A figure has been added to visualise programme development, data collection, and endpoints.
(Page 11, line 339-342)

Reviewer 2

1. Please include practical recommendations for sustaining the approach.
Response: Thank you for highlighting the importance of sustainability. Practical multi-level recommendations addressing this aspect have been added in Box 1 and referenced in both the Abstract and Discussion.
(Abstract: Page 2, lines 84–85; Discussion: Page 26, line 690; page 27, lines 705-7113, lines 724-727; page 29, lines 780-794 Box 1)

We hope these revisions fully address all comments and improve the clarity and contribution of the manuscript.
Thank you again for your constructive feedback and for the opportunity to resubmit our work.
